# Single dose of intraoperative intravenous morphine for analgesia in children undergoing tonsillectomy: Randomized, double-blind clinical trial

**DOI:** 10.1016/j.bjorl.2020.09.007

**Published:** 2020-10-15

**Authors:** Marcus Cavalcante de Oliveira Araújo, Juliana Alves de Sousa Caixeta, Breno Fernandes Vilarinho, Melissa Ameloti Gomes Avelino

**Affiliations:** a Universidade Federal de Goiás, Goiânia, GO, Brazil; b Centro Universitário de Anápolis, Anapólis, GO, Brazil

**Keywords:** Tonsillectomy, Morphine, Child, Analgesia, Anesthesia

## Abstract

**Introduction:**

Children undergoing tonsillectomy have severe pain in the postoperative period. One of the pharmacological options for analgesia is opioids, such as morphine. However, the risks of adverse effects, such as increased recovery time from anesthesia and respiratory depression, can limit its use.

**Objectives:**

To evaluate the use of intraoperative intravenous morphine to reduce immediate postoperative pain in children undergoing tonsillectomy.

**Methods:**

In this double-blind randomized study, children aged 3–10 years were submitted to tonsillectomy, with or without adenoidectomy, and divided into two groups. Children in group M received 0.1 mg/kg of intravenous morphine during anesthetic induction, while those in the control group received conventional anesthesia without morphine. Postoperative pain perceptions were assessed at 30, 60, 120, 180 and 240 min after recovery from anesthesia, by the children themselves and also by their parents or guardians, using a facial pain scale.

**Results:**

A total of 57 children were included, 30 in the group with morphine and 27 in the group without morphine. According to the children themselves, the postoperative pain was less at the evaluations performed at 30 min after awakening from anesthesia (*p* =  0.023), while according to their parents/guardians, the pain was less intense in the evaluations performed at 30 (*p* =  0.002), 60 (*p* =  0.006) and 180 min (*p* =  0.007) after awakening. Moreover, postoperative analgesics were less requested by children in the morphine group. No observed side effects were associated with the use of morphine.

**Conclusion:**

A single dose of intravenous morphine during anesthetic induction reduced the intensity of immediate postoperative pain in children undergoing tonsillectomy, without increasing the time of awakening from anesthesia and with lower consumption of rescue analgesics.

## Introduction

Tonsillectomies are one of the most common procedures performed in children worldwide.^1^ Although the mortality rate is low, postoperative pain can be severe, even with the available assortment of analgesic methods,^2^, ^3^ since there is still no consensus on the ideal therapeutic strategy for pain control.^4^

Opioids are one of several effective pharmacological treatment options in reducing post-tonsillectomy pain, acting not only as analgesics,^5^ but also reducing the agitation often caused by inhalation anesthesia in children.^6^ However, the risk of respiratory depression, particularly in otorhinolaryngological surgery, may limit the use of some of these drugs, such as tramadol and codeine.^7^

Morphine is an opioid with a widely known analgesic potential, being prescribed also for the relief of postoperative pain in pediatrics. However, its associated risks may include increased postoperative anesthetic recovery time, excessive sleepiness, aspiration and hypoxia, especially in pediatric patients with comorbidities such as obesity or severe sleep apnea.^8^, ^9^, ^10^

Another challenging aspect of postoperative pain in children can be its measurement, as the pain experienced by them can be often difficult to quantify, especially in patients under five years of age.^11^ But despite the limitations of currently available methods, they are the ones used to manage adequate analgesia.^12^

The aim of this study was to evaluate the efficacy of intraoperative intravenous morphine in reducing postoperative pain in children undergoing tonsillectomy with or without adenoidectomy.

## Methods

This was a blind, randomized and controlled study, approved by the local ethics committee (CAAE 46153415.6.0000.5078 and CEP Opinion number: 1,252,240) and registered at REBEC (Brazilian Registry of Clinical Trials). The study occurred from June 2016 to June 2019, and included children aged 3–10 years old, classified as American Society of Anesthesiologists (ASA) physical status 1–2, with indication for tonsillectomy, with or without adenoidectomy, for the treatment of respiratory sleep disorder caused by obstruction due to adenoid enlargement, with over 70% obstruction at the nasal videoendoscopy and/or tonsil hypertrophy grades III or IV; or recurrent tonsillitis, defined as 7 confirmed infections in 1 year, or 5 confirmed infections per year for 2 years or still 3 infections a year for 3 years. It was estimated that with 27 patients in each group, the study would have an 80% power to detect clinically important differences between the groups at the faces pain scale (FPS), assuming a large effect size (Cohen’s d = 0.80), with a 5% significance level. The sample size was increased by 10% to compensate for possible losses over the followup period, resulting in a total size of 60 patients, who were randomized to both groups.

The children who met the inclusion criteria were treated with a same-day surgery (outpatient) protocol, and during the preanesthetic consultation, these children and their parents or guardians were invited to participate in the research, with minors and adults signing the free and informed consent form. After agreeing to participate, they were shown a validated pain assessment scale using faces (FPS - faces pain scale), for the purpose of familiarization,^13^ which uses children's drawings with facial pain expressions, representing graduations ranging from no pain to the greatest possible pain.

The exclusion criteria included comorbidities such as cardiopulmonary dysfunction, cerebral palsy, obesity, chronic use of analgesics, anticonvulsants or neuroleptics, complications related to anesthesia (severe laryngo- or bronchospasm, anaphylaxis and/or hemorrhage), history of hypersensitivity to anesthetics and need for medications in addition to those described in the protocol.

The tonsillectomy surgeries were performed using the dissection technique (cold knife surgery) and hemostasis with standard surgical sutures using catgut thread. General anesthesia was used, and the monitoring consisted of pulse oximetry, cardioscopy, non-invasive blood pressure measurement and capnography. Anesthetic induction was carried out with 6%–8% sevoflurane under mask and oxygen with FiO2 of 100%. After the venoclysis was started, intravenous administration of propofol (2 mg/kg) and fentanyl (0.5 µg/kg) was performed, with orotracheal intubation. The patients were then divided into two groups. The randomization list was previously generated through a computer program, with random letters (M for morphine or O for no drugs) placed in and distributed in opaque brown paper envelopes, sealed and sequentially numbered, which were kept secret until the intraoperative moment. Thus, those in group M received 0.10 mg/kg of intravenous morphine during the anesthesia induction period, whereas those in group O did not receive any additional medication. Dimenhydrinate (1.25 mg/kg), ondansetron (0.15 mg/kg), dexamethasone (0.10 mg/kg), dipyrone (30 mg/kg) and ranitidine (1 mg/kg) were also administered intravenously to all patients. The anesthesia was maintained with 2%–4% sevoflurane and 50% FiO_2_. All patients were anesthetized by the main investigator and the surgeries were performed by otorhinolaryngology resident physicians. Surgery duration was defined as the period between the first incision and the final hemostasis, the time of anesthesia as the time between the beginning of inhalation anesthesia and the time of extubation, and the time of awakening from anesthesia as the time of sevoflurane withdrawal up to tracheal extubation. A chronometer was used to record all times.

At the end of the procedure, the patients were extubated with intact protective reflexes (coughing and swallowing), spontaneous and effective breathing, crying and movement, and sent to the post-anesthetic recovery room (PACU), where they were continuously monitored for at least four hours by an anesthesiologist different from the one who performed the anesthesia, by the nursing team and also by the child's guardian, all of whom were blinded to the use of morphine. Thus, with the exception of the anesthesiologist who performed the anesthesia and drew the envelope, all other professionals and also the family members were blinded to the study groups. The main objective was pain assessment, which was performed using the SPF scale in the PACU, recording data at 30, 60, 120, 180 and 240 min after the subjects awoke from the anesthesia. The face pain scale (FPS) depicts seven faces, which are shown and scored, from one corresponding to the absence of pain status to the one that reflects the worst possible pain (0: no pain; 1: mild pain; 2: moderate pain; 3: pain ; 4: severe pain; 5: very severe pain; 6: worst pain possible). If according to the FPS scale, the child and also their parents/guardians indicated pain intensity greater than or equal to 2, analgesia was performed by the intravenous administration of 0.5–2 µg/kg of fentanyl and, if necessary, repeated. In these cases, these patients were excluded from the subsequent hourly assessments of pain level, avoiding the influence of this analgesic on the results. Supplemental oxygen was administered through a nasal catheter or oxygen mask if oxygen saturation decreased below 94%. As secondary outcomes, the use of rescue analgesics in the postoperative period and the occurrence of adverse events, such as nausea, vomiting, bleeding, excessive sleepiness, pruritus and respiratory depression, as well as any other unexpected symptoms or events, were recorded. The Aldrete-Kroulik scale was also used for discharge from the PACU.

The groups were compared regarding age, weight, duration of surgery and awakening time from anesthesia using the Mann-Whitney non-parametric test. Group M was compared to group O to determine any differences in pain perceptions over time, as reported by patients and their parents/guardians. In these comparisons, a mixed model and analysis of variance (ANOVA) of repeated measures was applied. The Kaplan-Meier estimator was used to construct the survival curves between the two groups to estimate the probability of survival of the need to use rescue. The comparisons were made using the log-rank test. The level of significance was set at *p* < 0.05 and statistical analyses were performed using the SAS® software, version 9.4 (SAS institute, Cary, NC).

## Results

**Figure 1:**
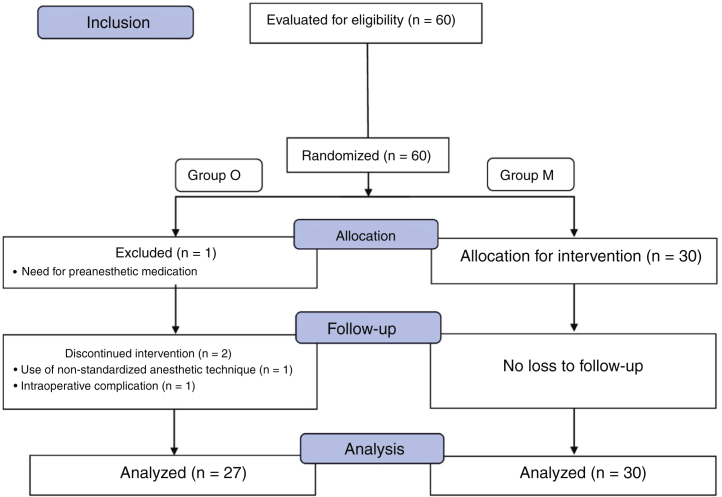
CONSORT Flowchart.

Sixty children were selected, 30 in group M and 30 in group O, with a mean age of 5.9 ± 2 years, 60% of which were males. Three patients, all belonging to the control group (group O), were excluded from the study (Fig. 1): one due to the need for pre-anesthetic medication, another for not following the standard anesthesia protocol, and the third for having severe laryngeal spasm during extubation. The groups did not differ significantly regarding the duration of surgery or the time to awaken from anesthesia, with the average time of the first being 43 min and the average time of the second being 12 min (Table 1).

**Table 1:** Number of participants, duration of surgery and time of awakening from the anesthesia.

Variables	Groups		*p*-value^a^
	O (mean ± SD)	M (mean ± SD)	
N. of participants (males./females)	27 (19/8)	30 (15/15)	
Duration of surgery (min.)	44.5 ± 13.8	42 ± 17.3	0.196
Time of awakening from the anesthesia (min.)	13.22 ± 5.35	12.10 ± 6.23	0.378

Independent *t*-test.

SD, Standard deviation.

a Mann-Whitney non-parametric test.

Pain was reported as more intense in the beginning of the postoperative period, both in group M and in group O, generally with progressively lower intensity until the fourth hour of evaluation, with a significant difference regarding the children's (*p* =  0.036) and parents/guardians (*p* =  0.003) evaluation scores in relation to group M and group O (Table 2).

**Table 2:** Mean values for the perception of pain by the children and their parents/guardians in the different postoperative times.

Moment of pain recording as reported by the child	Group O	Group M	p-value^a^	*p*-value
	(mean ± SD)	(mean ± SD)	O × M	Interaction between group and time
30 min	3.48 ± 0.39	2.23 ± 0.37	0.023^b^	0.036^b^
60 min	3.17 ± 0.43	2.70 ± 0.37	0.406	
120 min	2.43 ± 0.49	1.90 ± 0.37	0.394	
180 min	2.19 ± 0.51	1.45 ± 0.38	0.242	
240 min	1.83 ± 0.51	1.86 ± 0.38	0.962	

**Moment of pain recording as reported by the parents/guardians**				0003^b^
30 min	3.52 ± 0.32	2.17 ± 0.30	0.002^b^	
60 min	3.44 ± 0.35	2.17 ± 0.30	0.006^b^	
120 min	2.30 ± 0.40	1.33 ± 0.30	0.057	
180 min	2.58 ± 0.41	1.17 ± 0.31	0.007^b^	
240 min	1.87 ± 0.41	1.17 ± 0.31	0.180	

a The values for comparison between the groups were calculated using the mixed effects models of analysis of variance for repeated measures.

b *p* <  0.05.

**Figure 2:**
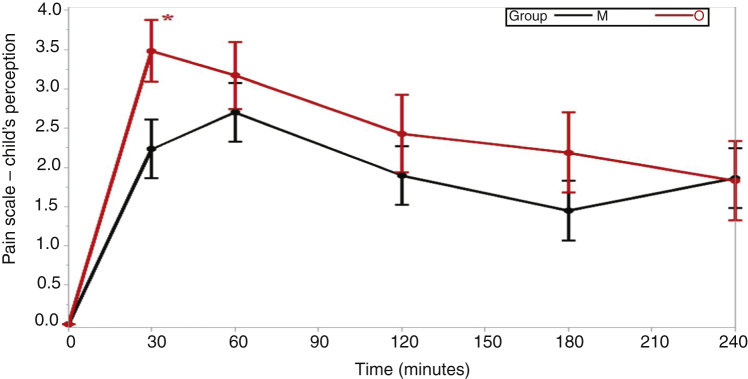
Comparison between the means of pain perceptions by the children in Groups M and O at times 30*, 120, 180 and 240 min (*p <  0.05).

**Figure 3:**
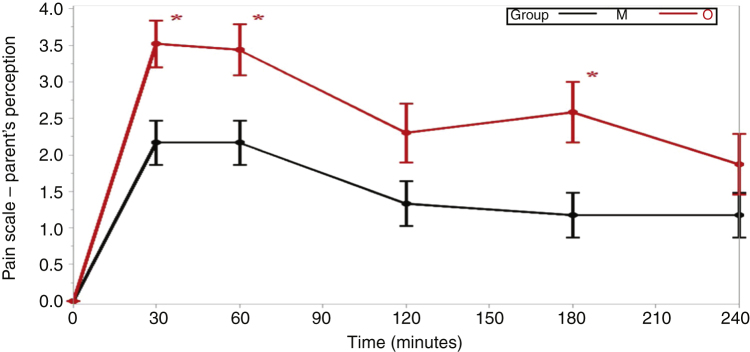
Comparison between the means of pain perceptions by the parents/guardians in Groups M and O at times 30*, 60*, 120, 180* and 240 min (*p <  0.05).

In the patients’ assessment, there was a report of less severe pain in group M 30 min after awakening from the anesthesia (*p* =  0.023), while all other assessments were similar (Table 2 and Fig. 2). When parents/guardians were asked to assess the patients' pain, their reports were significantly lower in group M in the evaluations performed at 30 (*p* =  0.002), 60 (*p* =  0.006) and 180 min (*p* =  0.007) after awakening from the anesthesia (Table 2 and Fig. 3).

Regarding the levels of pain reported within each of the groups, comparing what was reported by the child with that described by parents/guardians, the interactions between group and time were not significant for any of the groups (*p* =  0.751 and *p* =  0.918), demonstrating that the pain scale behavior over time of followup did not differ between the children's and parents' assessments for any time (Table 3).

**Table 3:** Comparison of the intensity of pain reported by the child and the parents/guardians of the same group in the postoperative period.

Interaction between groups and time (*p*-value)^a^	Group O		Group M	
	0.918 (mean ± SD)		0.751 (mean ± SD)	
	Children	Parents/Guardians	Children	Parents/Guardians
30 min	3.48 ± 0.41	3.52 ± 0.41	2.23 ± 0.30	2.17 ± 0.30
60 min	3.17 ± 0.43	3.46 ± 0.43	2.70 ± 0.30	2.17 ± 0.30
120 min	2.53 ± 0.49	2.40 ± 0.49	1.90 ± 0.30	1.33 ± 0.30
180 min	2.30 ± 0.50	2.70 ± 0.50	1.44 ± 0.31	1.17 ± 0.31
240 min	1.95 ± 0.50	1.98 ± 0.50	1.85 ± 0.31	1.17 ± 0.31

a The values were calculated using the mixed effects models of analysis of variance for repeated measures.

**Figure 4:**
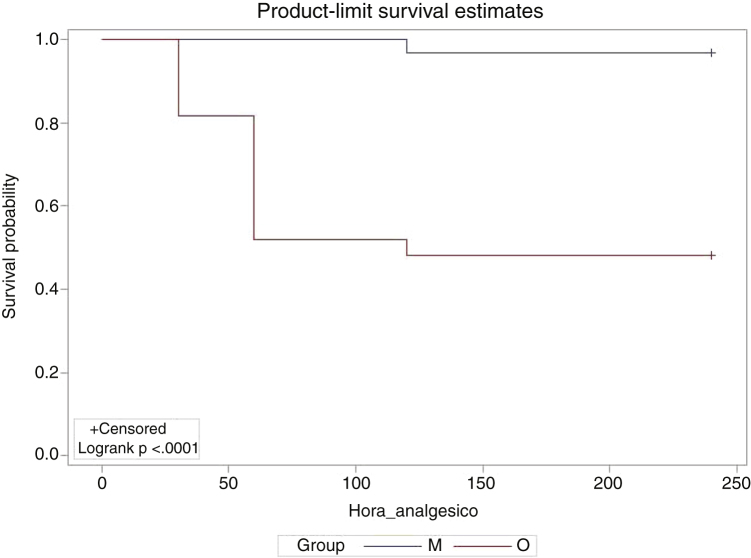
Need for rescue analgesics according to time in the Groups M and O (*p* < 0.0001).

The use of rescue analgesics was different in the groups, with 14 patients (51.85%) in group O who needed rescue analgesia in the postoperative period, on average after 53.57 min and with an average dose of 0.65 mcg/kg of fentanyl, while only one patient (3.33%) in group M required analgesics, at 120 min (Table 4). A Kaplan-Meier curve was constructed to compare the required time of rescue use, considering that a prospective follow-up was performed at different times (Fig. 4). Although there are restrictions regarding the amount of representative points according to the result of the log-rank test, the two survival curves differ from each other, showing that patients in group M show a higher probability of remaining without the need for rescue analgesia than patients in group O throughout the follow-up period (*p* <  0.0001). As for the adverse effects, only one patient in group O experienced nausea and vomiting, with no reports of other complications.

**Table 4:** Use of rescue analgesics in the postoperative period.

Analgesic	Grupo O	Grupo M	*p*-valor
No analgesics needed, n (%)	13 (48.15%)	29 (96.67%)	0.002^a^
Analgesics were needed	14 (51.85%)	1 (3.33%)	
Mean postoperative time (min.)	53.57 ± 24.05	120	
Mean dose (mcg/kg)	0.65	0.22	
Total of children	27	30	

a Independent *t*-test.

## Discussion

Children undergoing tonsillectomy experience significant pain and severe postoperative functional limitations for up to two weeks,^3^ usually more intense in the first few days and which are sources of great anxiety for parents and also for the children themselves. They include earache, refusal to eat, drink or take oral medications, edema, dysphagia and weight loss.^4^, ^5^ These conditions can be influenced by psychological distress and also by anesthesia emergence delirium in the immediate postoperative period.^6^

To avoid such effects and provide adequate postoperative analgesia, several strategies are used, such as pre-anesthetic medication, active participation of parents during the perioperative period and non-pharmacological and pharmacological measures. Some drugs can increase the risk of postoperative hypoxia, as in the case of opioids such as codeine (CYP2D6 polymorphisms)^7^ and tramadol, leading to their infrequent use. Additionally, they have other important side effects, which include nausea, vomiting, urinary retention, pruritus and constipation. Despite that, morphine can still be used with good results in post-tonsillectomy pain management,^8^, ^9^, ^10^ along with several other pharmacological options, such as non-steroidal anti-inflammatory drugs, dexamethasone, acetaminophen, ketamine, dexmedetomidine and infiltrative anesthesia.^14^, ^15^, ^16^

According to the results of the present study, intraoperative administration of 0.1 mg/kg of intravenous morphine in children undergoing tonsillectomy reduced the intensity of postoperative pain in the assessment made by the children themselves and also in the assessment of their parents/guardians, being effective in reducing pain after 30 min of awakening from the anesthesia, according to the patients, and after 30, 60 and 180 min, according to the parents/guardians, decreasing the use of rescue analgesic medication in the postoperative period and without prolonging the time of awakening from the general anesthesia in these patients or the adverse effects in the immediate perioperative period. It is important to note that there was still one patient in the group who received morphine who needed rescue analgesics in the postoperative period, indicating that the analgesia may not be adequate in all cases and reiterating the need to implement other alternative options for analgesic treatment.

Morphine is a narrow therapeutic index drug, with variations in the analgesic response and side effects, ranging from inadequate pain relief to the extreme of severe adverse results. Much of this inter-individual variability can be explained by single nucleotide polymorphisms in a subset of genes that encode proteins involved in pain mechanisms and opioids. The study of the association between this genotype and the clinical results associated with analgesia and adverse effects could identify, through genetic tests, which patients would benefit from its use,^17^, ^18^ even if this is still a distant reality. Therefore, the definition and titration of safer doses of morphine could minimize its adverse effects, meeting the patient’s individual needs and limiting the risk of overdose. 19, 20 It is always important to emphasize that the use of opioids to relieve pain can decrease the response to hypoxemia and increase the risk of respiratory depression, and that comorbidities such as sleep apnea, craniofacial disorders, bronchopneumonia, asthma, obesity and respiratory tract infections, combined with postoperative edema, can worsen their respiratory effects. Thus, an indispensable aspect, even with all the care and administration in standardized doses, is the recovery of these patients in the PACU, under constant monitoring.

No severe adverse events were observed in the outcome of this clinical trial. Some approaches used may have minimized the onset of these effects, such as the absence of pre-anesthetic medications like benzodiazepines or alpha-2 agonists, such as clonidine. The administration of morphine right after anesthetic induction, providing more time for its circulation and early therapeutic approach of its unwanted clinical effects, such as apnea, as well as the duration of surgery, which lasts on average 43 min, may also have contributed to the absence of these effects, since the surgeries were performed by residents and took longer than if they had been performed by more experienced surgeons. Another factor may have been the absence of children with major comorbidities, such as underweight, obesity or severe sleep apnea, which may lead to an increased risk of postoperative hypoxia.^21^ Another aspect in this study was the low occurrence of nausea and vomiting in the postoperative period (in only one patient), probably due to the pharmacological prevention strategy used in the intraoperative period. Another factor that can be discussed as a limitation of this clinical trial is the lack of research on other elements, such as genetic study or the patient’s gender, which could also interfere with the pharmacokinetics of morphine. Additionally, the dose of fentanyl in the anesthetic induction can also be considered low, since a higher dose would probably contribute to longer-lasting analgesia and lower pain scores during the first 30 min of the postoperative period, even considering that it was used in both groups. Therefore, although it may have limitations and be sporadic for a specific group of patients, this study suggests an reduction of postoperative pain in children who received morphine, regardless of other variables.

An important aspect of this type of study may be the difficulty with which children clearly demonstrate their pain level and their need for analgesics, since the ability to adequately assess pain usually begins to develop only around at the age of five.^11^ Young children who have not yet been enrolled in school are not used to questioning from strangers and are also inexperienced in providing classifications or quantitative estimates. These patients’ pain reports can also be affected by their perception of this classification consequences, because if they believe they might receive an injection for reporting pain, they may minimize their experiences or report a lower intensity of pain. The correlation with the evaluation carried out by the parents/guardians partially mitigates this problem. Despite this, pain assessments carried out by children’s self-report scales, such as those utilized in this study, are widely employed, as they are easier to understand, provide useful information and constitute a valid evaluation tool. The FPS is an easy-to-understand method, available in many countries and languages, and has been validated for use in the Portuguese language, which is why it is considered in this study, although several others have also been accepted and used worldwide.^12^ A possible limitation would be that it has not been validated for children under 4 years of age, although it was used in children aged 3 years and older in its original description, apparently with good understanding.^13^

Another important aspect is that the FPS scale is not valid to evaluate postoperative pain through parents and caregivers. However, no pain scale was standardized to be used by parents in the immediate postoperative period, up to 4 h, as in this study. Nevertheless, the modified FPS may be equivalent to the PPPM (Parents’ Postoperative Pain Measure), a scale particularly useful for parents to assess pain at home after outpatient surgery, for pain quantification in children after tonsillectomy.^22^

Postoperative analgesia should be considered even before the surgical stimulus, starting with the use of pre-anesthetic medications and parental monitoring, going through anesthetic-surgical techniques for a peaceful awakening and culminating with rescue analgesics in the postoperative period. However, the careful approach regarding analgesia in these children can often lead to excessive sedation in the postoperative period, increasing the risk of complications, such as respiratory depression. Some drugs used as pre-anesthetic medication, such as midazolam, clonidine or ketamine, can improve analgesia, but can also potentiate complications. Measures for the prevention and treatment of pain in this type of patient and the safety in the postoperative period, preventing adverse effects, can show a fragile balance, varying between the provided analgesic efficacy and the assured safety. In this study, we chose to avoid excessive sedation after the awakening from the anesthesia, and for this reason we do not use pre-anesthetic medications.

Studies with morphine, administered alone or at other doses, associated with other analgesic drugs or even in different age groups, can add more knowledge to those presented by this study.

## Conclusion

According to the results of this study, morphine administered intravenously and during the intraoperative period in children undergoing tonsillectomy reduces the intensity of immediate postoperative pain, without increasing the time of awakening from anesthesia and with less consumption of rescue analgesics in the postoperative period, without significant adverse effects.

## Conflicts of interest

The authors declare no conflicts of interest.
